# Adherence to the Mediterranean diet and risk of gastric cancer: a systematic review and dose–response meta-analysis

**DOI:** 10.3389/fnut.2023.1259453

**Published:** 2023-09-08

**Authors:** Qin Zhu, Long Shu, Feng Zhou, Li-Peng Chen, Yu-Liang Feng

**Affiliations:** ^1^Department of Digestion, Zhejiang Hospital, Hangzhou, Zhejiang, China; ^2^Department of Nutrition, Zhejiang Hospital, Hangzhou, Zhejiang, China

**Keywords:** Mediterranean diet, gastric cancer, systematic review, meta-analysis, dose–response, epidemiology

## Abstract

**Background:**

Despite growing evidence for the association of adherence to the Mediterranean diet with gastric cancer risk, the results remain inconclusive. The purpose of this systematic review and meta-analysis was to summarize the evidence from previous observational studies and assess the potential association between adherence to the Mediterranean diet and risk of gastric cancer using a dose–response meta-analysis.

**Methods:**

A comprehensive literature search for all observational studies published up to June 30, 2023 was conducted using the databases of PubMed, ISI Web of Science, EBSCO, China National Knowledge Infrastructure (CNKI) and Wanfang Data. The pooled relative risks (RRs) and 95% confidence intervals (CIs) were calculated for the highest versus the lowest categories of Mediterranean diet score in relation to gastric cancer risk, using random-effects models. The Cochran’s *Q* test and *I*-squared (*I^2^*) statistic were used to detect the sources of heterogeneity among the included studies.

**Results:**

Overall, 11 studies (five cohort and six case–control studies) with a total number of 1,366,318 participants were included in the final analysis. Combining 14 effect sizes from 11 studies revealed that compared with the lowest category, the highest adherence to the Mediterranean diet was associated with a 29% reduction in the risk of gastric cancer (RR:0.71; 95%CI:0.59–0.84, *p* < 0.001). In addition, linear dose–response analysis showed that each 1-score increment in Mediterranean diet score was associated with a 5% lower risk of gastric cancer (RR:0.95; 95%CI: 0.94–0.96, *p <* 0.001). Stratified analysis showed a significant association between adherence to the Mediterranean diet and risk of gastric cancer in case–control studies (RR = 0.44;95%CI:0.32–0.61, *p* < 0.001), and a marginally significant association in prospective cohort studies (RR = 0.88; 95%CI: 0.79–0.98, *p* = 0.024), respectively. At the same time, a more significant association between Mediterranean diet and reduced risk of gastric cancer was observed in other countries (RR = 0.28; 95%CI:0.16–0.49, *p* < 0.001) than in Western countries (RR = 0.75; 95%CI:0.64–0.88, *p* = 0.001).

**Conclusion:**

Our results demonstrate that high adherence to the Mediterranean diet is associated with 29% reduced risk of gastric cancer. Further large prospective studies and randomized controlled trials are warranted to confirm our findings.

## Introduction

Gastric cancer, also known as stomach cancer, is one of the most common malignancies worldwide, and its incidence and mortality rate has steadily declined over the last one-half century (1). According to the latest estimates released by GLOBOCAN, in 2020, gastric cancer remains the fifth most commonly diagnosed cancer and fourth leading cause of cancer-related mortality globally, with >1 million new case and an estimated 769,000 deaths (2). Notably, the incidence of gastric cancer is significantly higher in Eastern Asia compared to North America and Europe (2, 3). In China, gastric cancer has been the third leading cause of death in all cancers, and 0.33 million new cases and 0.37 million deaths occurring in 2020 (3). This trend reflects the urgency and necessity of implementing effective strategies for the prevention of gastric cancer. As far as we know, increasing evidence has shown that gastric cancer is induced by the combined synergistic effects of genetic factors, *Helicobacter pylori* (*H. pylori*) infection, cigarette smoking, alcohol intake, and dietary factors (4).

Over the past decades, diet has been recognized as a leading contributor to gastric cancer (5). Mounting epidemiological studies have mostly examined the correlations between intakes of individual foods (6), nutrients (7) or overall dietary patterns (8) and the risk of gastric cancer. In the meantime, the latest report by the World Cancer Research Fund/American Institute for Cancer Research (WCRF/AICR) states that high consumption of alcoholic drinks and foods preserved by salting are associated with an increased risk of gastric cancer (9). The Mediterranean diet, characterizing by a high intake of fruits, vegetables, nuts, legumes, whole grains and extra-virgin olive oil; a moderate intake of poultry, fish and alcohol; and a low intake of red and processed meats (10), represented a healthy dietary pattern usually consumed in the populations bordering the Mediterranean sea (11). Up to date, accumulating epidemiological evidence has suggested the beneficial role for high adherence to the Mediterranean diet on certain chronic non-communicable diseases, such as cardiovascular diseases, non-alcohol fatty liver disease and some types of cancers (12–14). Still, little is known with regard to the relationship between *a priori* defined the Mediterranean diet adherence and risk of gastric cancer. Nonetheless, based on the characteristics of the Mediterranean diet, it could be an ideal dietary pattern to reduce the risk of gastric cancer.

The relationship between adherence to the Mediterranean diet and cancers has been a focus for researchers in recent years (15). Only a few studies have so far explored the association between adherence to the Mediterranean diet and risk of gastric cancer (16–26), but the conclusions of previous studies are not entirely consistent. Several case–control studies have shown a significant inverse relationship between adherence to the Mediterranean diet and gastric cancer risk (16, 17, 19, 20, 22, 24, 26), while other studies showed the null association (18, 21, 23, 25). For example, in a hospital-based case–control study by Amiry et al., greater adherence to the Mediterranean diet was associated with a lower odds of gastric cancer (OR:0.17; 95%CI: 0.03–0.80) (16). However, no significant association was observed between adherence to the alternate Mediterranean diet and gastric cancer risk in the Multiethnic cohort study (25). Besides, to our knowledge, a recent systematic review and meta-analysis (Morze et al., 2021) reported a reduction of 30% in the incidence of gastric cancer for the highest adherence to the Mediterranean diet (27). Nevertheless, this meta-analysis included only seven articles, and neither dose–response relationship or subgroup analyses were performed in the main analysis. Since then, several new observational studies on this topic have been published (16, 19, 20, 25). Thus, to address the current gaps in knowledge regarding the relationship between adherence to the Mediterranean diet and gastric cancer risk, we performed a comprehensive systematic review and dose–response meta-analysis to summarize the current evidence of observational studies published from inception up to June 2023.

## Materials and methods

This study was carried out in accordance with the standards of the Preferred Reporting Items for Systematic Reviews and Meta-Analysis (PRISMA) statement (28). Moreover, the protocol of this systematic review and meta-analysis has not been registered in the International Prospective Register of Systematic reviews.

### Literature search strategy

We carried out a comprehensive search of articles published up to June 30, 2023 using PubMed, ISI Web of Science, EBSCO, CNKI and Wanfang Data with no restrictions in language or publication date. The following terms were used in our search: {(“stomach cancer”[all fields] OR “stomach neoplasm” [all fields] OR “gastric cancer”[all fields] OR “gastric neoplasm”[all fields] OR “cancer of the stomach”[all fields]) AND (“MedDiet”[all fields] OR “Mediterranean diet” [all fields] OR “Mediterranean”[all fields] OR “Dietary pattern”[all fields] OR “dietary score”[all fields] OR “dietary adherence”[all fields])}. In addition, a manual search in the reference lists from the retrieved articles or reviews or meta-analyses was performed to find the potentially eligible studies. All of these steps were accomplished by two independent reviewers (ZQ and SL), and any disagreements with article selection were resolved through discussion with Y-LF. Our selection criteria was based on the PECOS (e.g., participant, exposure, comparison, outcome, and study design) framework, which is shown in Supplementary Table S1.

### Studies included criteria

Two independent reviewers (QZ and LS) carried out an initial screening of all titles and abstracts from retrieved articles to identify eligible studies that should be included in the analysis. Any disagreements were settled by discussion or in consultation with the third reviewer (Y-LF). When all reviewers agreed, the full-text articles were reviewed against inclusion and exclusion criteria for the present systematic review and updated meta-analysis. To be included in the present meta- analysis, articles had to meet the following criteria: (1) observational studies (i.e., case–control and cohort studies) conducted in adult population (aged ≥18 years); (2)considered adherence to the Mediterranean diet as the exposure; (3) assessed the association between adherence to the Mediterranean diet and risk of gastric cancer; (4) provided estimates of RRs, HRs, ORs with their corresponding 95%CIs; (5) If the data in retrieved article lacked sufficient detail, the corresponding author of the original study was contacted by email; (6) gastric cancer diagnoses were confirmed by clinical interviews, or self-report on a previous physician-made diagnosis of gastric cancer. Moreover, studies were excluded if they fulfilled one of the following criteria: (1) unrelated articles; (2) non-observational studies, e.g., reviews, editorials, case reports and conference letters; (3) animal, cell culture, and *in vitro* studies; (4) studies not reported as HRs, RRs or ORs with 95%CIs.

### Data extraction

After completing selection of all eligible studies, two independent authors (LS and FZ) extracted the following information: first author’s last name, publication year, study design, country, sample size, number of gastric cancer cases, mean age/age range, follow-up time (cohort studies), components of the Mediterranean diet score, methods of dietary assessment, reported risk estimates (HR/OR/RR) and the corresponding 95%CIs and confounding factors that were adjusted for in the multivariate analyses. Any discrepancies and disagreements about data extraction were resolved by consensus or discussion with the third author (Y-LF).

### Quality assessment of included studies

The Newcastle-Ottawa Scale (NOS) was used to evaluate the overall quality of the included studies in the present study (29). We assigned 0 ~ 9 “stars” to each study based on three major domains: the population selection (maximum of 4 stars), comparability of the groups (maximum of 2 stars), and outcome/exposure assessment (maximum of 3 stars). For this analysis, we considered that an NOS score ≥ 7 indicated high methodological quality (10). Differences were resolved by consensus with a third author (ZQ).

### Statistical analysis

The reported HRs in the primary studies were considered as equal as RRs (30). ORs were converted into RRs using the formula:RR = OR/[(1-P_0_) + (P_0_*OR)], in which P_0_ indicates the incidence of the outcome of interest in the non-exposed group (31). Log-transformed RRs with their corresponding standard errors (SEs) were obtained using risk ratios (ORs, HRs, and RRs and corresponding 95% CIs) which were previously extracted for the relationship between adherence to the Mediterranean diet and risk of gastric cancer. Heterogeneity across studies was tested using the Cochran’s *Q* test and the *I^2^* statistic. If *p-*values of Cochran’s *Q*-test ≤0.10 or *I*^2^ ≥ 50% indicated an absence of heterogeneity among studies, and the random-effects model (DerSimonnian and Laird method) was used to pool the RRs and 95%CIs of the highest versus the lowest category of Mediterranean diet in relation to gastric cancer. Otherwise, the fixed effect model is used (32). If significant between-study heterogeneity was observed, sensitivity and subgroup analyses would be performed to further find out the source of heterogeneity. In our analyses, subgroup analyses were performed based on sex (male or female), study design (cohort or case–control studies), anatomical site (gastric cardia or non-cardia), study area [Western countries or other countries (Afghanistan and Jordan)], mean age (≥50 or < 50), and sample size (<5,000 or >5,000). Sensitivity analysis was performed by removing one study at a time, and to clarify whether the results were robust or sensitive to the influence of a single study. If ≥10 comparisons were available, publication bias was evaluated through the visual inspection of the funnel plot and quantified by the Begg’s test and Egger’s test, respectively (33). If there was evidence of publication bias, we further evaluated the number of missing studies in a meta-analysis by the application of the Duval and Tweedie trim- and- fill method and recalculated the pooled estimates with the addition of those missing studies (34). Finally, we also performed a dose–response meta-analysis to estimate the RRs for each 1-score increment in Mediterranean diet adherence. A two-stage GLST model based on generalized least squares method was used to test the potential linear or non-linear dose–response association between adherence to a Mediterranean diet and risk of gastric cancer. We modeled Mediterranean diet scores by using restricted cubic splines with 3 knots at fixed percentiles (10, 50, and 90%) of the distribution. A *p-*value for curve linearity or non-linearity was computed by testing the null hypothesis that the coefficient of the first spline is equal to the second spline. All the mentioned data analyses were performed using STATA, version 12.0 (StataCorp, College Station, Texas, USA). A *p-value* less than 0.05 (two-tailed) was considered statistically significant.

## Results

### Search results

**Figure 1:**
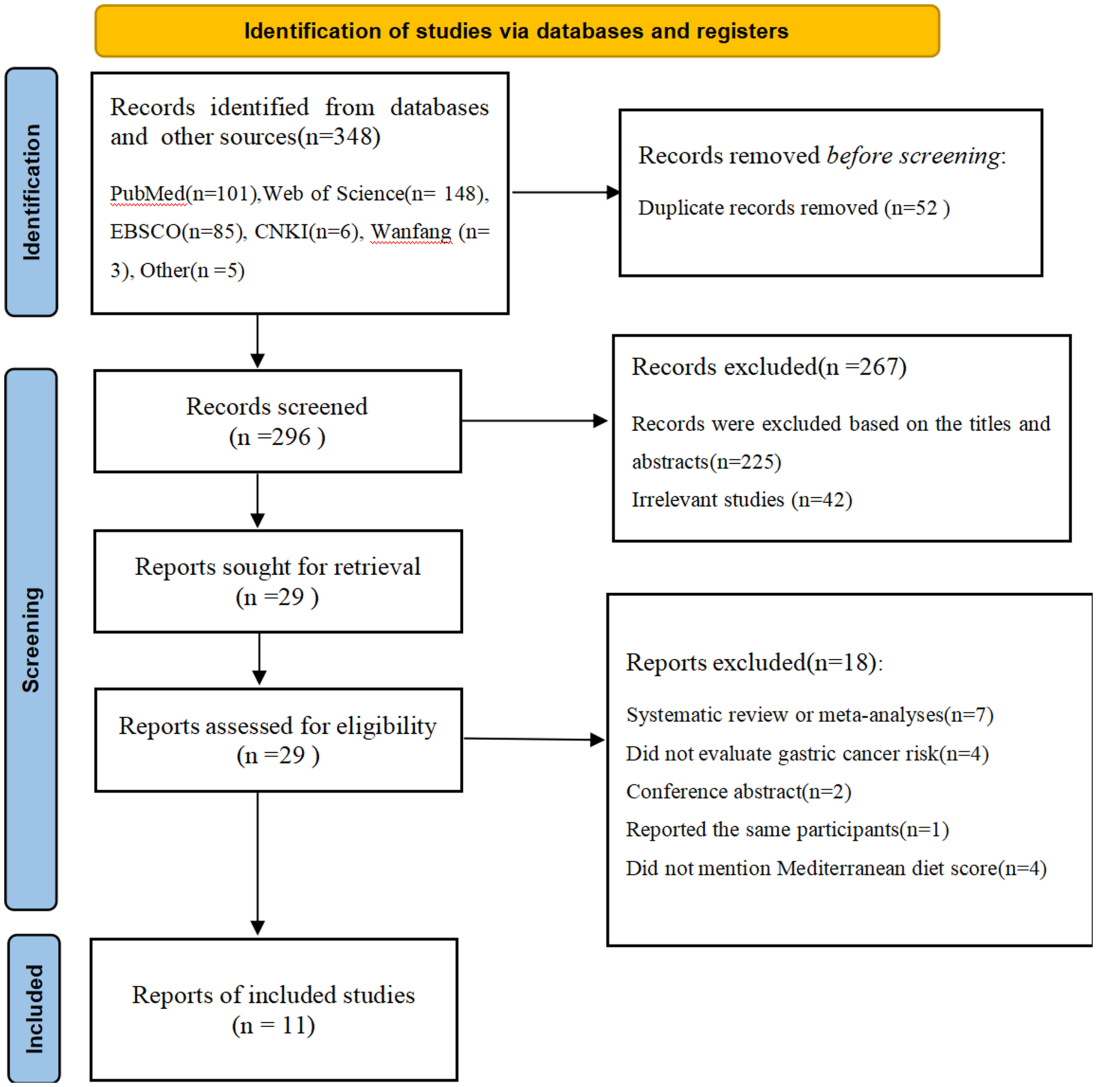
Flow chart of the process of study selection.

Figure 1 indicates the process of study selection. Our initial search yielded 348 potential articles, of which 52 were duplicates. Of the remaining 296 articles, we excluded 225 articles on the basis of the titles and/or abstracts; 42 articles on the basis of irrelevant studies. Then, after reading the full-text versions of the remaining 29 articles, 18 articles were excluded for the following reasons: 7 were systematic review or meta-analyses, 4 did not evaluate gastric cancer risk; 2 were conference abstracts; 1 reported the same participants; 4 did not mention Mediterranean diet score. Finally, 11 studies met the eligibility criteria and were included in this meta-analysis.

### Study characteristics

Table 1 shows the general characteristics of all included studies. A total of 11 studies, including 5 prospective cohort (18, 21, 23, 25, 26) and 6 case–control (16, 17, 19, 20, 22, 24) studies, met the inclusion criteria and was included in this study. These included studies were published between 2010 and 2023. The age of participants ranged from ages 18 to above. Two of the included studies were carried out in the United States (21, 25), two in Spain (19, 22), two in Italy (17, 24), one in Afghanistan (16), one in Sweden (23), one in Netherlands (18), one in Jordan (20), and one study in European countries (26). Thus, United States, Spain, Italy, Sweden, Netherlands and European countries were regarded as Western countries, while Afghanistan and Jordan were regarded as other countries. In all included studies, dietary intake was measured by using an FFQ (16–26). For outcome assessment, four studies had used cancer registries (18, 21, 23, 25), two studies used medical records (20, 24), and five studies used pathology reports (16, 17, 19, 22, 26). All included studies had an NOS score ≥ 7, which were of high quality (16–26). Among these studies, three studies (18, 21, 25) separately reported the relationship between adherence to Mediterranean diet and gastric cardia adenocarcinoma and non-cardia adenocarcinoma. The number of study participants varied between 270 and 494,968.

**Table 1:** Characteristics of included studies on the relationship between adherence to the Mediterranean diet and risk of gastric cancer.

Author Publication Year	Country	Study design	Total number of participants	Mean age/age range	Dietary assessment method	Adjustment or matched for in analyses	Effect sizes OR/RR (95%CI)
Amiry et al. 2022 (16)	Afghanistan	Case–control	270 (90 cases)	20-75y	FFQ	Age, sex, physical activity, Marriage status (married/not married), Kebab food (yes/no), smoking usage (yes/no), tooth brushing (do not brush, brush), job (former and worker, others), education (non-university graduate, university graduate), alcohol usage (yes/no), BMI (categorical).	0.17 (0.03–0.80)
Praud et al. 2014 (17)	Italy	Case–control	3,627 (999 cases)	19-80y	FFQ	Age, sex, study, year of interview, education, body mass index, tobacco smoking, family history, and total energy intake.	0.57 (0.45–0.70)
Schulpen et al.2019 (18)	Netherlands	Cohort	12,085 (777 cases)	55-69y	FFQ	Age at baseline (years), sex (men, women), cigarette smoking status (never, former, current), cigarette smoking frequency (cigarettes smoked per day, centered), cigarette smoking duration (years, centered), body mass index (kg/m^2^), daily energy intake (kilocalories), alcohol consumption (g/day), highest level of education (primary school or lower vocational, secondary school or medium vocational, higher vocational or university), non-occupational physical activity (≤30, >30- ≤ 60, >60- ≤ 90, >90 min per day), family history of esophageal cancer (for esophageal cancer subtypes; no, yes), and family history of gastric cancer (for gastric cancer subtypes; no, yes)	Gastric cardia adenocarcinoma:0.86 (0.71–1.04); gastric non-cardia adenocarcinoma:0.83 (0.73–0.93)
Álvarez-Álvarez et al. 2021 (19)	Spain	Case–control	3,394 (354 cases)	20-85y	FFQ	Sex, age, education, family history of gastric cancer (first degree), tobacco status, total energy consumed, BMI (the year before diagnosis), consumption of NSAIDs, and total time of physical activity as fixed terms and area of residence	0.32 (0.22–0.46)
Tayyem et al. 2022 (20)	Jordan	Case–control	486 (172 cases)	≥18y	FFQ	Age, marital status, BMI, education, smoking, physical activity, family history, and energy (Kcal).	0.21 (0.11–0.42)
Li et al. 2013 (21)	United States	Cohort	494,968 (954 cases)	50-71y	FFQ	Age, sex, race, smoking, alcohol intake, education, BMI, vigorous physical activity, usual activity, and total energy intake	Gastric cardia adenocarcinoma: 1.10 (0.76–1.61);gastric non-cardia adenocarcinoma: 0.75 (0.52–1.09)
Castelló et al. 2018 (22)	Spain	Case–control	3,092 (271 cases)	20-85y	FFQ	Sex, age, education, BMI, family history of gastric cancer, physical activity (METs), smoking status, caloric intake, and alcohol intake as fixed effects and province of residence	0.53 (0.34–0.82)
Bodén et al.2019 (23)	Sweden	Cohort	100,881 (163 cases)	40-60y	FFQ	Energy intake, BMI, physical activity, smoking, educational status	0.85 (0.69–1.03)
Stojanovic et al.2017 (24)	Italy	Case–control	446 (223 cases)	≥18y	FFQ	Sex, tobacco smoking, and total energy intake.	0.70 (0.61–0.81)
Acuna et al.2023 (25)	United States	Cohort	176,752 (1,043 cases)	45-75y	FFQ	Age at cohort entry, sex, self-identified race and ethnicity (including birthplace), a family history of gastric cancer, education, smoking status, pack-years of cigarette smoking, aspirin use status, and total energy intake.	Gastric cardia adenocarcinoma: 1.56 (0.95–2.54); Gastric distal adenocarcinoma:0.99 (0.78–1.26)
Buckland et al.2010 (26)	European countries	Cohort	485,044 (449cases)	35-70y	FFQ	Center and age and adjusted for sex, BMI, educational level, smoking status, cigarette smoking intensity, and total energy intake	0.67 (0.47–0.94)

BMI, body mass index; FFQ, food frequency questionnaire; NSAIDs, non-steroidal antiinflammatory drugs; “Schulpen 1, Li 1 and Acuna1” indicate gastric cardia adenocarcinoma; “Schulpen2, Li 2 and Acuna12” indicate gastric non-cardia adenocarcinoma.

### Adherence to the Mediterranean diet and gastric cancer incidence

**Figure 2:**
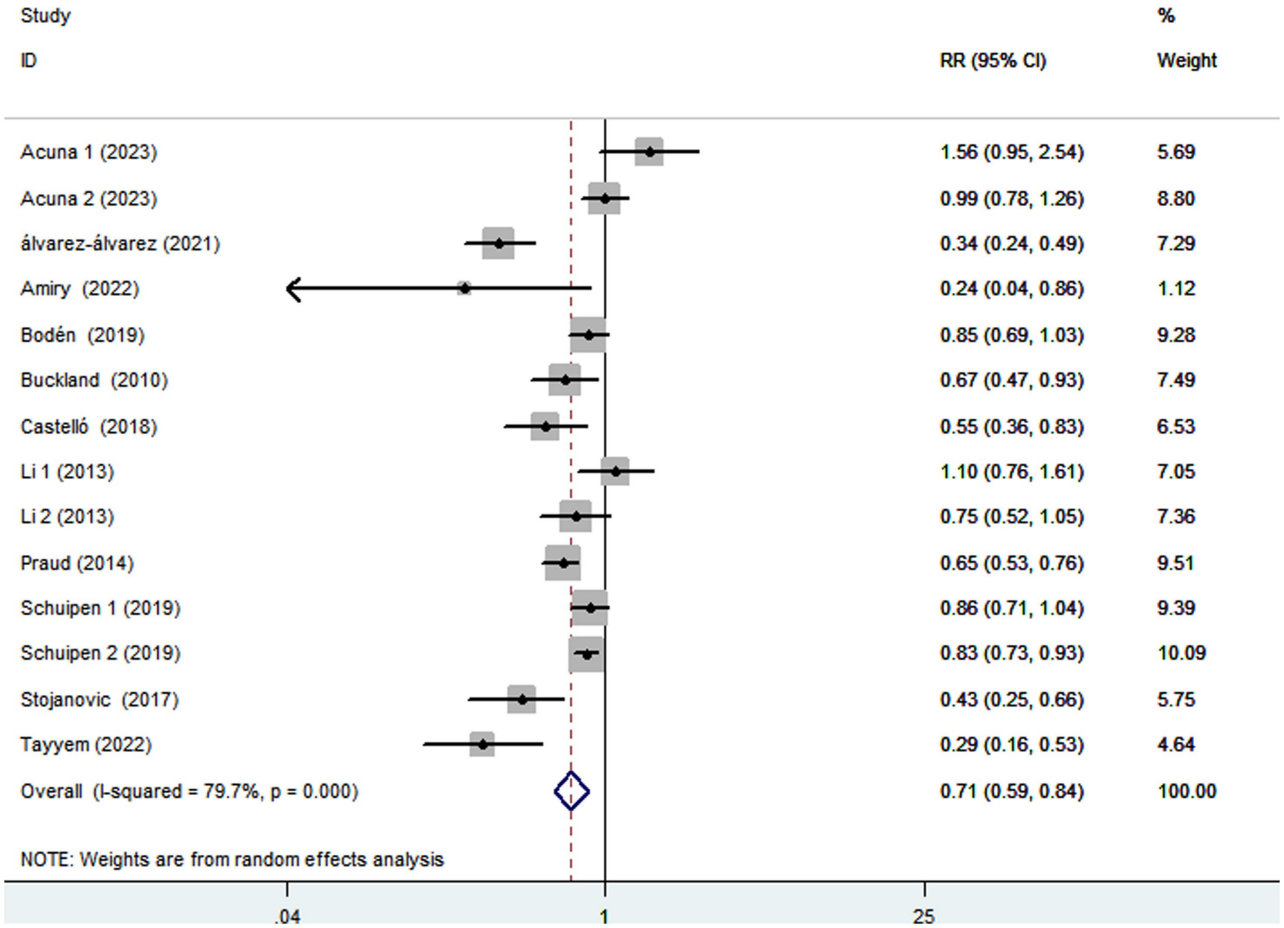
Forest plot of the association between adherence to the Mediterranean diet and risk of gastric cancer.

Eleven articles comprising 5,708 gastric cancer cases and 1,366,318 participants, were included to assess the link between adherence to the Mediterranean diet and the risk of gastric cancer. Combining 14 effect sizes from 11 studies, Figure 2 showed the evidence of a decreased risk of gastric cancer in the highest compared with the lowest categories of Mediterranean diet (RR = 0.71; 95%CI:0.59–0.84, *p* < 0.001). There was evidence of substantial heterogeneity between studies (*I*^2^ = 79.7%, *p* < 0.001), and therefore the effect was assessed using the random-effects model.

### Dose–response analysis

**Figure 3:**
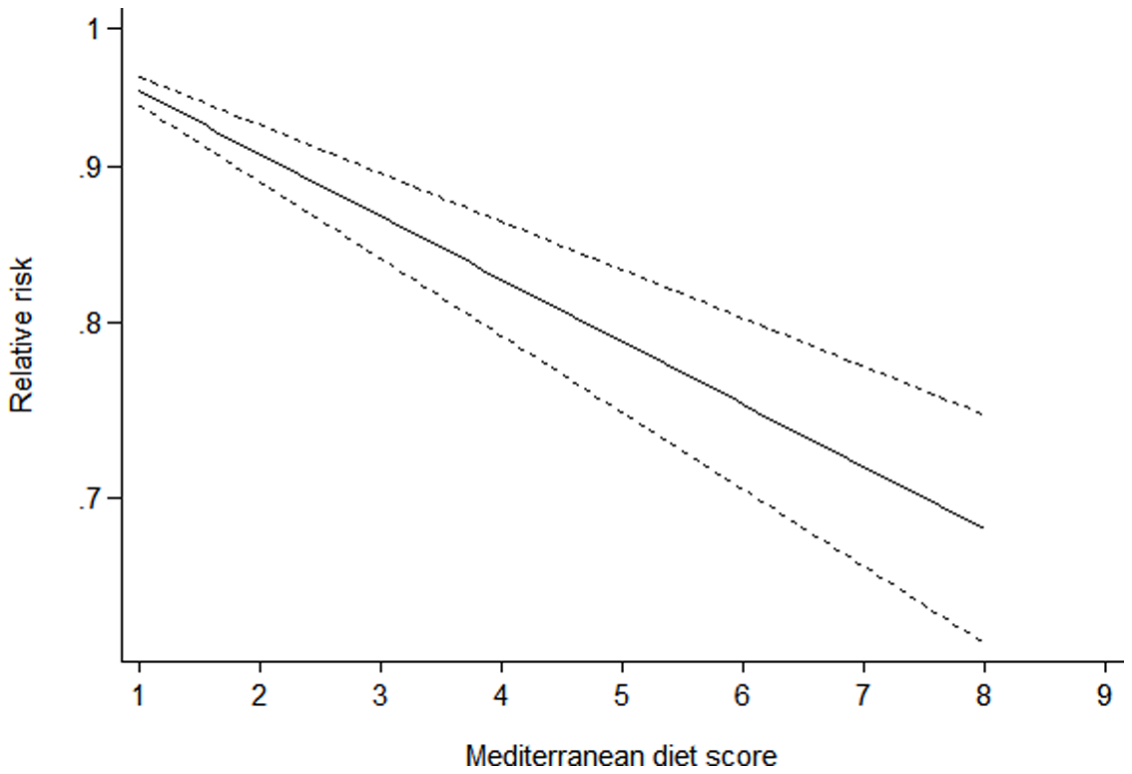
Forest plot of the association between each 1-score increment in Mediterranean diet and risk of gastric cancer.

**Figure 4:**
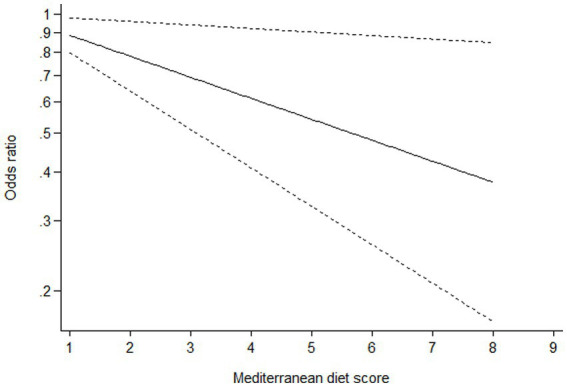
Dose-response association between adherence to the Mediterranean diet and risk of gastric cancer in the analysis of six case-control studies.

**Figure 5:**
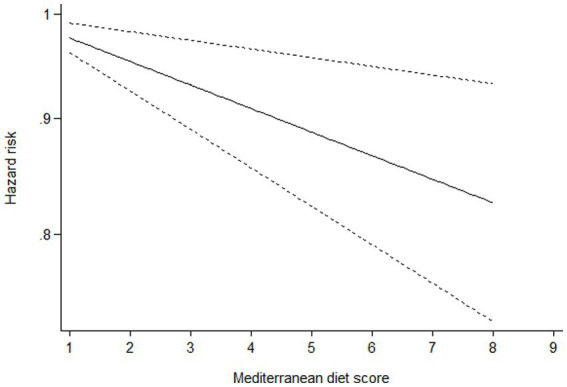
Dose-response association between adherence to the Mediterranean diet and risk of gastric cancer in the analysis of four cohort studies.

Ten studies (16–22, 24–26) containing 6 case–control studies with Mediterranean diet scores on the same scale (0–9) were included in the dose–response analysis for gastric cancer risk. A linear dose–response analysis showed that each 1-score increment in Mediterranean diet score was associated with a 5% lower risk of gastric cancer (RR = 0.95;95%CI:0.94–0.96, *p <* 0.001; *P*_non-linearity_ = 0.330) (Figure 3). The analysis of six case–control studies showed a positive linear relationship between Mediterranean diet and risk of gastric cancer (OR = 0.89; 95%CI:0.80–0.98; *p* = 0.018; *P*_non-linearity_ = 0.382) (Figure 4). In addition, the analysis of four cohort studies also showed a positive linear relationship between Mediterranean diet and risk of gastric cancer (HR = 0.98, 95%CI:0.96–0.99; *p* = 0.002; *P*_non-linearity_ = 0.538) (Figure 5).

### Subgroup analyses

Given the high heterogeneity of this study (*I*^2^ = 79.7%; *p* < 0.001), subgroup analyses were performed to explore the potential sources of heterogeneity (Table 2). In this study, subgroup analyses were carried out basing on study design (cohort or case–control studies), study area (Western countries or other countries), mean age (≥50 or <50), sample size (<5,000 or >5,000), anatomical site (gastric cardia or non-cardia) and sex (male or female). When we conducted analyses separately by study design, results showed an inverse relationship between Mediterranean diet and risk of gastric cancer in cohort studies (RR = 0.88; 95%CI: 0.79–0.98, *p* = 0.024), with less evidence of heterogeneity between studies (*p =* 0.115; *I*^2^ = 39.6%). In contrast, there was also significant association between Mediterranean diet and decreased risk of gastric cancer in case–control studies (RR = 0.44; 95%CI: 0.32–0.61, *p* < 0.001), with evidence of heterogeneity (*p =* 0.005; *I*^2^ = 69.8%). For study area, we found a significant inverse association between adherence to Mediterranean diet and gastric cancer risk in other countries (RR = 0.28; 95%CI: 0.16–0.49, *p* < 0.001). There was no evidence of heterogeneity between studies (*p =* 0.822; *I*^2^ = 0.0%). When the results were stratified by mean age, we found a significant inverse association between Mediterranean diet and gastric cancer risk in age ≥ 50 and age < 50 (for age ≥ 50: RR = 0.70; 95%CI: 0.57–0.87, *p* = 0.001 and for age < 50: RR = 0.71; 95%CI: 0.53–0.95, *p* = 0.022). The heterogeneity was most apparent in age ≥ 50 (*p <* 0.001; *I*^2^ = 82.5%). For sample size, we found a significant inverse relationship between Mediterranean diet and gastric cancer risk in the subgroups of sample size > 5,000 (RR = 0.88; 95%CI: 0.79–0.98, *p* = 0.024). However, the heterogeneity was less (*p* = 0.115, *I*^2^ = 39.6%). In addition, a protective association was found between Mediterranean diet and gastric cancer risk in the studies with sample size<5,000 (RR = 0.44; 95%CI: 0.32–0.61, *p* < 0.001), and there was significant heterogeneity (*p* = 0.005, *I*^2^ = 69.8%). We also performed stratified analysis based on sex, and results showed the inverse association between Mediterranean diet and gastric cancer risk in male and female (0.77 vs. 0.91). however, there was no evidence of heterogeneity in female (*p =* 0.604; *I*^2^ = 0.0%).

**Table 2:** Subgroup analyses for the relationship between adherence to Mediterranean diet and stomach cancer risk.

Study characteristic	No. of studies	RR (95%CI)	Heterogeneity	
			*I*^2^(%)	*P*
All	11	0.71 (0.59–0.84)	79.7	<0.001
Study design				
Case–control	6	0.44 (0.32–0.61)	69.8	0.005
Cohort	5	0.88 (0.79–0.98)	39.6	0.115
Study area				
Western countries	9	0.75 (0.64–0.88)	78.6	<0.001
Other countries	2	0.28 (0.16–0.49)	0.0	0.822
Mean age				
≥50	8	0.70 (0.57–0.87)	82.5	<0.001
<50	3	0.71 (0.53–0.95)	65.7	0.054
Sample size				
>5,000	5	0.88 (0.79–0.98)	39.6	0.115
<5,000	6	0.44 (0.32–0.61)	69.8	0.005
Anatomical site				
Gastric non-cardia	7	0.74 (0.60–0.91)	79.7	<0.001
Gastric cardia	7	0.77 (0.61–0.98)	66.6	0.006
Sex				
Male	5	0.77 (0.68–0.88)	67.7	0.015
Female	5	0.91 (0.86–0.96)	0.0	0.604

RR, relative risk; CI, confidence interval; Afghanistan and Jordan were defined as other countries.

### Publication bias and quality assessment

Funnel plots showed little evidence of asymmetry (Supplementary Figure S1) and therefore no evidence of publication bias existed (highest compared with lowest category of Mediterranean diet: Begg’s test, *p* = 0.324; Egger’s test, *p* = 0.161). Due to no evidence of publication bias, we could not perform the trim and fill analysis to adjust the pooled effect estimates. All included studies in our analyses have received a NOS score ≥ 7, and these studies were regarded to be of high quality (16–26) (Table 3).

**Table 3:** Mediterranean diet and risk of gastric cancer: Assessment of Study Quality.

Studies	Selection				Comparability		Outcome			Score
	1	2	3	4	5A	5B	6	7	8	
Cohort										
Schulpen et al. 2019 (18)	*	*	*	*	*	*	*	*	*	9
Li et al. 2013 (21)	*	*	*	*	*		*	*	*	8
Bodén et al. 2019 (23)	*	*	*	*	*	*	*	*	*	9
Acuna et al. 2023 (25)	*	*	*	*	*	*	*	*	*	9
Buckland et al. 2010 (26)	*	*	*	*	*		*	*	*	8
Case–control										
Amiry et al. 2022 (16)	*			*	*	*	*	*	*	7
Praud et al. 2014 (17)	*			*	*	*	*	*	*	7
Álvarez-Álvarez et al. 2021 (19)	*	*	*	*	*		*	*		7
Tayyem et al. 2022 (20)	*	*		*	*	*	*	*		7
Castelló et al. 2018 (22)	*	*	*	*	*		*	*		7
Stojanovic et al.2017 (24)	*			*	*	*	*	*	*	7

*For case–control studies, 1 indicates cases independently validated; 2, cases are representative of population; 3, community controls; 4, controls have no history of gastric cancer; 5A, study controls for the most important factor; 5B, study controls for additional factors, e.g., cigarette smoking body mass index, total energy intake; 6, ascertainment of exposure by secure record or blinded interview or record; 7, same method of ascertainment used for cases and controls; and 8, the same for cases and controls. For cohort studies, 1 indicates exposed cohort truly representative; 2, non-exposed cohort drawn from the same community; 3, ascertainment of exposure by secure record (e.g., surgical records) or structured interview; 4, outcome of interest was not present at start of study; 5A, study controls for the most important factor; 5B, study controls for additional factor(s); 6, assessment of outcome is based on independent blind assessment or record linkage; 7, follow-up long enough (≥5 years) for outcomes to occur; and 8, adequacy of follow up of cohorts (all participants complete follow up or > 90% participants complete follow up).

### Sensitivity analyses

Based on the results of sensitivity analysis, the pooled results of Mediterranean diet on gastric cancer did not materially change when removing any single studies in the main analysis (RR ranged between 0.65 and 0.76) (Supplementary Figure S2).

## Discussion

To our knowledge, this is the latest systematic review and meta-analysis to evaluate the association between adherence to the Mediterranean diet and gastric cancer risk with 1,366,318 participants and 5,708 cases of gastric cancer. The current systematic review and meta-analysis of 11 epidemiological studies shows that greater adherence to the Mediterranean diet was significantly associated with a 29% reduction in the risk of gastric cancer. In addition, the dose–response analysis also shows that each 2-score increment in Mediterranean diet was associated with a 6% reduction in the risk of gastric cancer. In a meantime, sensitivity analysis revealed that the summary effect of Mediterranean diet on gastric cancer was not substantially modified by excluding a certain study. Collectively, the results of this systematic review and meta-analysis provide further scientific evidence supporting the adoption of adherence to the Mediterranean diet for the primary prevention of gastric cancer.

Thus far, many epidemiological studies have examined different dietary patterns in relation to the risk of gastric cancer (16, 17, 35–37). However, most previous studies have mainly used *a posteriori* methods to evaluate the association between certain dietary patterns (e.g., healthy/prudent and Western patterns) and gastric cancer (35–37). In contrast, the impact of *a priori*-defined dietary pattern, e.g., Mediterranean diet on gastric cancer has rarely been investigated (16, 17). Still, the impact of Mediterranean diet on gastric cancer has also attracted much attention. Also of note, current research findings on the relationship between adherence to the Mediterranean diet and gastric cancer risk are in debate. For example, in a prospective cohort of the National Institutes of Health (NIH)-AARP Diet and Health Study, Li et al., found that aMED scores were not significantly associated with gastric cardia or non-cardia adenocarcinomas (21). On the contrary, in a hospital-based case–control study, Amiry et al., found that greater adherence to Mediterranean diet might be associated with a lower odds of gastric cancer (16). These inconsistent findings in previous studies might be explained by the differences in study design and study populations. In our analyses, adherence to the Mediterranean diet was associated with a reduced risk of gastric cancer. Our findings were aligned with previous meta-analyses (8, 38), which showed that the healthy/prudent dietary patterns were associated with a decreased risk of gastric cancer. Similarly, in a hospital-based case–control study by Toorang et al., high adherence to the DASH dietary pattern was associated with a 54% decrease risk of gastric cancer (OR:0.46; 95%CI: 0.26–0.83) (39). In fact, the healthy/prudent and DASH dietary patterns share some similarities with the Mediterranean diet, such as high consumption of vegetables, fruits and whole grains. Several previous studies have reported the favorable effect of fruit and vegetables intake on gastric cancer (40, 41). Furthermore, a recent systematic review and meta-analysis of Mediterranean diet and risk of cancer also reported that highest adherence to the Mediterranean diet was related to lower risk of gastric cancer (27). However, the above mentioned meta-analysis only included seven articles and dose–response relationship and subgroup analyses were not conducted in their analyses. Also, due to the limited number of studies, Morze et al. did not performed the publication bias. In this context, identifying the link between adherence to the Mediterranean diet and gastric cancer risk through a dose–response meta-analysis would appear to have value. Whilst current evidence on the relationship of Mediterranean diet with gastric cancer risk remains inconsistent, several probable mechanisms have been put forward to explain the observed beneficial association. First, it is commonly known that vegetables and fruits are two main components of the Mediterranean diet. As reported in previous studies, fruits and vegetables intake have a favorable effect on lowering the risk of gastric cancer (40, 41). As far as we know, vegetables, fruits and whole grains are a rich source of dietary fiber. A previous meta-analysis based on 21 observational studies showed that dietary fiber intake was inversely associated with the risk of gastric cancer (42). Meanwhile, prior studies have also demonstrated that high intake of dietary fiber was associated with a lower risk for insulin resistance, an important risk factor for gastric cancer (43). Second, Mediterranean diet often contains high amounts of fruits, vegetables, legumes, and these foods are rich in antioxidants, e.g., vitamin C, vitamin E and other carotenoids compounds. Previous studies have clearly shown that these antioxidants can neutralize reactive oxygen species and protect against free radical damage involved in carcinogenesis (44). For instance, earlier studies have shown that vitamin C can protect cells from oxidative DNA damage, thereby blocking carcinogenesis (45). In addition, Mei et al. also reported that high intake of vitamin C can not only ameliorate gastric mucosal inflammation by scavenging reactive oxygen species, but also inhabit the growth of *H. pylori*, an important risk factor for stomach cancer (46). Third, fruits and vegetables are rich sources of folate. A previous study showed that folate was necessary for synthesis of thymine and play an important role in the synthesis, repair, and methylation of DNA, and thus preventing carcinogenesis (47). Fourth, nuts and legumes that provide a good source of polyphenols, including flavonoids and proanthocyanidins, are also recommended in the Mediterranean diet. The growing body of scientific evidence indicates that flavonoids can prevent cancer through inactivation of carcinogens, inhibition of cell proliferation, enhancement of DNA repair processes, and reduction in oxidative stress (48). Fifth, it is well-known that the Mediterranean diet is characterized by lower intake of red and processed meats. A recent dose–response meta-analysis found that red and processed meats could increase the risk of gastric cancer (6). In fact, processed meats often contain high amount of salt, nitrates or nitrites, and nitrosamine compounds, which have been thought to be carcinogenic (6, 49). Finally, high adherence to the Mediterranean dietary pattern is significantly associated with reduced risks of weight gain and obesity, which are established risk factors for gastric cancer (50). All together, the aforementioned these mechanisms may account for the beneficial association between adherence to the Mediterranean diet and gastric cancer.

Although we found a significant inverse association between adherence to the Mediterranean diet and gastric cancer, statistical heterogeneity between studies was significant (*I*^2^ = 79.7%; *p* < 0.001). As far as we know, inter-study heterogeneity is common in previous meta-analyses (38, 42, 51), but exploring the potential sources of high heterogeneity is necessary. In this study, subgroup analyses were carried out basing on study design (cohort or case–control studies), study area (Western countries or other countries), mean age (≥50 or < 50), sample size (<5,000 or >5,000), anatomical site (gastric cardia or non-cardia) and sex (male or female). The results of subgroup analyses showed that high statistical heterogeneity might mainly be attributed to the differences in study design, study area, sample size and sex. When we performed analyses separately by study area and sex, the heterogeneity decreased from 79.7 to 0.0%. Similarly, when we analyzed study design and sample size separately, results suggested that the heterogeneity decreased from 79.7 to 39.6%. Although the significant heterogeneity found between included studies cannot be fully explored by any of the above variables, there are several possible explanations for this high heterogeneity. First, all included studies assessed dietary intakes using FFQs, in which recall bias is unavoidable. Meanwhile, the only five cohort studies were included in this meta-analysis, which somewhat limits the significance of the pooled results. Second, despite all of the included studies have adjusted for potential confounders, we cannot fully exclude the effect of residual or unmeasured confounding factors on the observed relationship. Consequently, we inevitably have a high level of heterogeneity when pooling studies. Third, given the differences in the Mediterranean diet among different populations, despite HRs or RRs or ORs were all from the highest category (taking the lowest category as the reference), different studies may define Mediterranean diet slightly differently, resulting in significant heterogeneity. Additionally, the results were combined from retrieved studies conducted in different populations, resulting in significant heterogeneity. Finally, significant heterogeneity still persisted in the subgroup analyses, suggesting the presence of other unmeasured confounding factors.

### Strengths and limitations

The current meta-analysis has several notable strengths. First, this is the first comprehensive systematic review and dose–response meta-analysis so far evaluating the association between adherence to the Mediterranean diet and gastric cancer risk. Our findings add the available evidence and underline the importance of supporting the people in adhering to the Mediterranean diet for prevention of gastric cancer. Second, we used a comprehensive search strategy of five main databases which identified all the observational studies available. Third, the cases of gastric cancer have been diagnosed through view of cancer registry or medical records or pathological records by clinicians, avoiding misdiagnosis. Fourth, no signs of publication bias were evident in the funnel plot, and the Begg’s and Egger’s tests for publication bias were non-significant. Thus, our results were relatively stable. Fifth, the quality assessment showed that all of the included studies in this meta-analysis were of high quality, and the reported ORs/RRs/HRs were multivariate and adjusted for some known confounders. Finally, the adequate number of included studies allowed us to perform subgroup analyses for some important risk factors, e.g., study design, sex and anatomical site. Besides, the dose–response analysis was performed to strengthen the relationship between adherence to the Mediterranean diet and gastric cancer risk. Despite the above-mentioned strengths, some limitations of the current meta-analysis should also be acknowledged in interpretation of our findings. First, 6 out of 11 studies included in current study used the case–control design, which is more prone to recall and selection biases. In addition, owing to the observational nature of included studies, the possibility of residual bias from unmeasured or unknown confounders remains. Hence, further prospective cohort studies or randomized controlled trials are needed to confirm the role of Mediterranean diet in the prevention of gastric cancer. Second, in all of the included studies, dietary intake was measured through FFQs, which carried an inherent recall bias. Meanwhile, the levels of the highest and the lowest categories of Mediterranean diet scores were inconsistent in the included studies, which might have attenuated the true association. Third, although some known confounding factors have been adjusted in all eligible studies, the existence of residual confounding from unmeasured factors cannot be completely excluded. Fourth, significant heterogeneity was observed in our analyses. Even though we performed subgroup and sensitivity analyses to explore the potential sources of heterogeneity, we could not ascertain and explain the sources of inter-study heterogeneity sufficiently. Finally, this meta-analysis had a geographical restriction, as the majority of included studies came from the United States and European countries. Hence, the generalization of our findings to other populations should be taken with caution. Further large prospective studies are needed in other populations with different environmental conditions and dietary preferences.

## Conclusion

In conclusion, this study showed that high adherence to the Mediterranean diet was significantly associated with a reduced risk of gastric cancer. Our findings add to the current evidence that healthy dietary pattern, like the Mediterranean diet, could offer a practical strategy in the prevention of gastric cancer. To further reinforce the significance of our findings, prospective cohort studies and clinical trials are required to corroborate the observed association between high adherence to the Mediterranean diet and reduced risk of gastric cancer.

## Data availability statement

The original contributions presented in the study are included in the article/Supplementary material, further inquiries can be directed to the corresponding author.

## Author contributions

QZ: conceptualization, formal analysis, funding acquisition, writing – original draft. LS: data curation, formal analysis, funding acquisition, methodology, writing – review and editing. FZ: data curation, writing – review and editing. L-PC: methodology, writing – review and editing. Y-LF: supervision, writing – review and editing.

## Funding

The author(s) declare financial support was received for the research, authorship, and/or publication of this article. This study was supported by the National Natural Science Foundation of China (grant number: 82004040), Medical and Health research fund project of Zhejiang Province (no. 2022KY006) and Traditional Chinese Medicine Research Project of Zhejiang (Nos. 2020ZB009, 2021ZB010). The sponsors played no role in the study design, data collection, or analysis, or decision to submit the article for publication.

## Conflict of interest

The authors declare that the research was conducted in the absence of any commercial or financial relationships that could be construed as a potential conflict of interest.

## Publisher’s note

All claims expressed in this article are solely those of the authors and do not necessarily represent those of their affiliated organizations, or those of the publisher, the editors and the reviewers. Any product that may be evaluated in this article, or claim that may be made by its manufacturer, is not guaranteed or endorsed by the publisher.

## Abbreviations

AICR: American Institute for Cancer Research
BMI: body mass index
CIs: confidence intervals
DASH: dietary approaches to stop hypertension
FFQ: food frequency questionnaire
HRs: Hazards ratios
IARC: International Agency for Research on Cancer
ORs: odds ratios
RR: relative risks
PRISMA: preferred reporting items for systematic reviews and meta-analyses
WHO: World Health Organization
WCRF: World Cancer Research Fund
